# Psychotic Symptomatology in Adolescentes witch Autistic Spectrum Disorder

**DOI:** 10.1192/j.eurpsy.2022.1097

**Published:** 2022-09-01

**Authors:** L. Mallol Castaño, R. Paricio Del Castillo, P. Del Sol Calderón

**Affiliations:** Hospital Universitario Puerta de Hierro, Psiquiatría Infanto-juvenil, Majadahonda, Spain

**Keywords:** autistic spectrum disorder, comorbity, Psychosis, Adolescents

## Abstract

**Introduction:**

INTRODUCTION: Patients with autistic spectrum disorders may exhibit symptoms that can also appear in psychotic disorders, such as isolation and difficulties in social interaction. In addition, these patients may also present psychotic symptoms throughout their lives, sometimes difficult to differentiate from the patient’s own idiosyncrasies.

**Objectives:**

OBJECTIVES: To deepen the knowledge of the comorbidity of autism spectrum disorders, in particular psychosis, as well as the differential diagnosis in order to establish an adequate treatment plan and a multidisciplinary approach.

**Methods:**

METHODS: A detailed description is given of two cases of adolescents diagnosed with Autistic Spectrum Disorder who have presented time-limited psychotic symptomatology in the last year. In addition, a literature search was conducted on the comorbidity of psychosis in patients with ASD.

**Results:**

RESULTS: Both patients have required several hospital admissions to psychiatric units when they have had psychotic decompensations and psychopharmacological treatment with antipsychotics has been initiated.

**Conclusions:**

CONCLUSIONS: Patients with autistic spectrum disorders have a wide comorbidity. Psychosis can appear in these patients, often starting in adolescence, a time when social demands increase and patients can become decompensated. They require rapid, multi-level intervention.

**Disclosure:**

No significant relationships.

